# The combination of body mass index and fasting plasma glucose is associated with type 2 diabetes mellitus in Japan: a secondary retrospective analysis

**DOI:** 10.3389/fendo.2024.1355180

**Published:** 2024-02-14

**Authors:** Xiaofang Zhao, Tianci Yao, Bei Song, Haohui Fan, Ting Liu, Guangyu Gao, Kun Wang, Weilin Lu, Chengyun Liu

**Affiliations:** Department of Geriatrics, Union Hospital, Tongji Medical College, Huazhong University of Science and Technology, Wuhan, China

**Keywords:** type 2 diabetes mellitus, body mass index, fasting plasma glucose, insulin resistance, Japan

## Abstract

**Background:**

Body mass index (BMI) and fasting plasma glucose (FPG) are known risk factors for type 2 diabetes mellitus (T2DM), but data on the prospective association of the combination of BMI and FPG with T2DM are limited. This study sought to characterize the association of the combination of BMI and FPG (ByG) with T2DM.

**Methods:**

The current study used the NAGALA database. We categorized participants by tertiles of ByG. The association of ByG with T2DM was expressed with hazard ratios (HRs) with 95% confidence intervals (CIs) after adjustment for potential risk factors.

**Results:**

During a median follow-up of 6.19 years in the normoglycemia cohort and 5.58 years in the prediabetes cohort, the incidence of T2DM was 0.75% and 7.79%, respectively. Following multivariable adjustments, there were stepwise increases in T2DM with increasing tertiles of ByG. After a similar multivariable adjustment, the risk of T2DM was 2.57 (95% CI 2.26 - 2.92), 1.97 (95% CI 1.53 - 2.54) and 1.50 (95% CI 1.30 - 1.74) for a per-SD change in ByG in all populations, the normoglycemia cohort and the prediabetes cohort, respectively.

**Conclusion:**

ByG was associated with an increased risk of T2DM in Japan. The result reinforced the importance of the combination of BMI and FPG in assessing T2DM risk.

## Introduction

Concomitantly with the change of lifestyles and dietary habits, the incidence of type 2 diabetes mellitus (T2DM) is increasing year by year. By 2045, this diabetes epidemic is expected to 700 million worldwide (1). Since the increasing prevalence of diabetes and subsequent economic burden, diabetes has emerged as one of the most important public health issues (2). Identifying people at higher risk of developing T2DM and risk factors of T2DM could inform screening, prevention and earlier intervention.

Many studies have indicated that insulin resistance (IR) is the major metabolic disorder in the early stages of T2DM development (3–5). Thus, early recognition of IR is crucial. Traditional methods used to evaluate IR mainly include positive hyperinsulin ⁃ glucose clamp and homeostasis model assessment (6). However, they are complicated and time-consuming, and have been performed only under limited conditions. Triglyceride glucose (TyG) index-related parameters, including the TyG index and triglyceride glucose–body mass index (TyG-BMI), have been widely studied and reported as valuable biomarkers in the identification of various diseases, such as non-alcoholic fatty liver disease (7–9), metabolic syndrome (10, 11), heart failure (12), stroke (13), hyperuricemia (14). In recent times, TyG and TyG-BMI have been attracted much attention as new surrogate markers for the assessment of IR and T2DM (15–19). And besides, studies have found that TyG-BMI is of greater diagnostic value than TyG (19, 20), which shows that body mass index (BMI) has also played a significant role. BMI and fasting plasma glucose (FPG) are two well known risk factors for T2DM (21, 22). However, the relationship of the combination of BMI and FPG with T2DM is not clear. Accordingly, we proposed a new index-ByG (defined as ln [1/2 BMI (kg/m^2^) × FPG (mg/dL)]) based on the formula of TyG (defined as ln [1/2 TG (mg/dL) × FPG (mg/dL)]). Using a population-based cohort, we evaluated the association of ByG with T2DM.

## Materials and methods

### Data source

All the data analyzed in this study were obtained from The DATADRYAD database (http://www.Datadryad.org/). The raw data could be freely download from the dryad data package (Okamura, Takuro et al. (2019)) (23).

### Study cohort

This study was a cohort study that included 15,464 subjects in Japan. All the participants come from the NAGALA (NAfld in the Gifu Area, Population-based Longitudinal Analysis) database, which aimed to investigate risk factors for chronic diseases. All inclusion and exclusion criteria are detailed in the literature of the data source (23). The exclusion criteria for the study of raw data included: 1) T2DM at baseline or fasting plasma glucose ≥ 6.1 mmol/L; 2) missing data; 3) known liver disease; 4) ethanol consumption (> 60 g/day for men and 40 g/day for women); medication usage (23). The study was approved by the Ethics Committee of Murakami Memorial Hospital in Japan. Informed consent was obtained from participants in the study (23).

### Exposure and covariates

BMI = weight (kg) divided by height^2^(m). TyG = Ln [(FPG (mg/dL)/2) × TG (mg/dL)] (8). TyG-BMI = BMI × TyG (8). The study exposure was ByG. The formula for calculating the index: ByG = Ln [1/2 BMI (kg/m^2^) × FPG (mg/dL)].

The sociodemographic characteristics included age, sex, habit of exercise, alcohol consumption (non, light, moderate, heavy), and smoking status (never, past, current). The laboratory results were as follows: BMI, waist circumference (WC), alanine aminotransferase (ALT), aspartate aminotransferase (AST), gamma-glutamyl transferase (GGT), total cholesterol (TC), triglycerides (TG), high-density lipoprotein cholesterol (HDL), FPG, glycosylated haemoglobin (HbA1c), systolic blood pressure (SBP), and diastolic blood pressure (DBP). Criteria for fatty liver diagnosis by abdominal ultrasonography were as follows: hepatorenal echo contrast, liver brightness, deep attenuation, and vascular blurring (23).

### Outcome

Newly developed T2DM was either diagnosed following the American Diabetes Association criteria [FPG ≥ 7 mmol/l or HbA1c ≥ 6.5%] (24) or was based on self-report (25).

### Statistical analyses

The total cohort was divided into the normoglycemia (defined as FPG < 100mg/dL or HbA1c < 5.7%) group and the prediabetes (defined as FPG≥100mg/dL or HbA1c level of 5.7% to 6.4%) group (26). For normally distributed continuous variables, one-way analysis of variance was used to analyze the differences among groups, expressed as mean ± standard deviation. For non-normally distributed continuous variables, differences across groups were analyzed using Kruskal-Wallis test and expressed as median with interquartile ranges. Categorical variables were compared by the chi-square test and expressed as proportions.

Characteristics of participants were described by ByG tertiles. Then, a univariate analysis model was applied to explore the relation between the baseline characteristics and T2DM. Moreover, Cox proportional hazards models and restricted cubic spline (RCS) analysis were conducted to assess the association of the levels of ByG with T2DM. Additionally, we performed receiver operating characteristic (ROC) curve analysis and informativeness analysis (27) to examine the potential diagnostic value of ByG in T2DM. Finally, a stratified study and interaction analysis between ByG and T2DM were identified in different subgroups by grouping sex, fatty liver, smoking status (never, past, current), alcohol consumption (non, light, moderate, heavy), and habit of exercise. All statistical analyses in our study were executed by EmpowerStats (www.empowerstats.com, X&Y solutions, Inc., Boston MA) and R statistical software (http://www.R-project.org).

## Results

### Characteristics of individuals by tertiles of ByG

**Figure 1:**
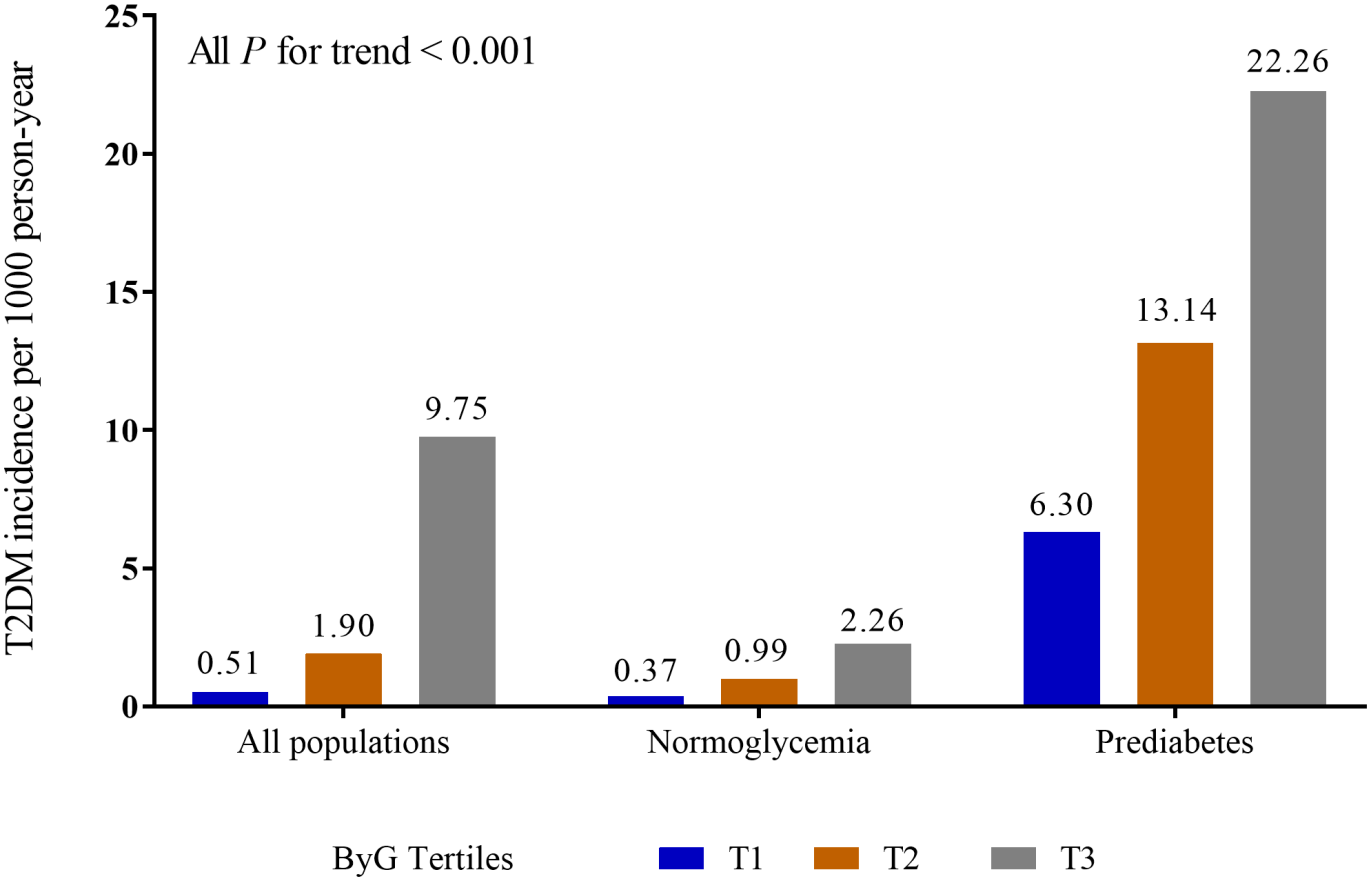
Incidence of T2DM in different populations. ByG, body mass index glucose; T2DM, type 2 diabetes mellitus.

A total of 15,464 participants were included in the cohort study. There were 11,806 participants in the normoglycemia cohort and 3,658 participants in the prediabetes cohort. **Table 1** presented the characteristics of the study stratified by ByG tertiles in all populations. Compared with subjects in the lowest tertile of ByG, those in the highest tertile were more likely to be older, male, smoking (past or current), drinking (light or moderate or heavy), have fatty liver, have no habit of exercise, have higher BMI, WC, ALT, AST, GGT, TC, TG, HbA1c, FPG, SBP, DBP and lower HDL. Additionally, the ByG highest tertile group had the highest T2DM incidence, with 9.75 cases per 1000 person-years in all populations, 2.26 cases per 1000 person-years among the normoglycemia cohort and 22.26 cases per 1000 person-years among the prediabetes cohort (**Figure 1**).

**Table 1:** Baseline characteristics of the study sample according to ByG tertiles.

Variable	ByG			*P*-value
	Tertile 1	Tertile 2	Tertile 3	
	(5.88 - 6.84)	(6.84 - 7.00)	(7.00 - 7.71)	
Sample size	5155	5154	5155	
Age, yrs	41.84 ± 8.78	44.16 ± 8.81	45.12 ± 8.78	<0.001
BMI, kg/m2	19.22 ± 1.49	21.85 ± 1.40	25.29 ± 2.57	<0.001
WC, cm	68.43 ± 5.52	76.08 ± 5.60	84.90 ± 7.15	<0.001
ALT, IU/L	14.00 (11.00-17.00)	16.00 (13.00-21.00)	22.00 (16.00-31.00)	<0.001
AST, IU/L	16.00 (13.00-19.00)	17.00 (14.00-20.00)	19.00 (15.00-23.00)	<0.001
GGT, IU/L	12.00 (10.00-15.00)	15.00 (12.00-21.00)	21.00 (15.00-32.00)	<0.001
TC, mg/dl	189.90 ± 32.19	197.33 ± 32.54	207.39 ± 33.16	<0.001
HDL, mg/dl	64.00 ± 15.26	56.55 ± 14.71	49.07 ± 12.90	<0.001
TG, mg/dl	47.00 (34.00-66.00)	65.00 (46.00-93.00)	94.00 (64.00-139.00)	<0.001
HbA1c, %	5.10 ± 0.30	5.14 ± 0.31	5.27 ± 0.33	<0.001
FPG, mg/dl	86.99 ± 5.82	93.08 ± 5.46	98.83 ± 5.68	<0.001
SBP, mmHg	106.41 ± 12.60	114.17 ± 12.99	122.91 ± 14.46	<0.001
DBP, mmHg	66.09 ± 8.78	71.26 ± 9.44	77.39 ± 10.04	<0.001
Sex				<0.001
Women	3811 (73.93%)	2106 (40.86%)	1117 (21.67%)	
Men	1344 (26.07%)	3048 (59.14%)	4038 (78.33%)	
Fatty liver				<0.001
No	5089 (98.72%)	4638 (89.99%)	2996 (58.12%)	
Yes	66 (1.28%)	516 (10.01%)	2159 (41.88%)	
Smoking status				<0.001
Never	3819 (74.08%)	2915 (56.56%)	2297 (44.56%)	
Past	548 (10.63%)	1016 (19.71%)	1388 (26.93%)	
Current	788 (15.29%)	1223 (23.73%)	1470 (28.52%)	
Alcohol consumption				<0.001
No	4397 (85.30%)	3855 (74.80%)	3553 (68.92%)	
Light	419 (8.13%)	660 (12.81%)	679 (13.17%)	
Moderate	271 (5.26%)	448 (8.69%)	641 (12.43%)	
Heavy	68 (1.32%)	191 (3.71%)	282 (5.47%)	
Habit of exercise				<0.001
No	4300 (83.41%)	4166 (80.83%)	4289 (83.20%)	
Yes	855 (16.59%)	988 (19.17%)	866 (16.80%)	

Continuous variables are presented as mean (SD) or median (25th, 75th percentile), and categorical variables are presented as number (percentage).

ByG, body mass index glucose; BMI, body mass index; WC, waist circumference; ALT, alanine aminotransferase; AST, aspartate aminotransferase; GGT, gamma-glutamyl transferase; TC, total cholesterol; HDL, high-density lipoprotein cholesterol; TG, triglycerides; HbA1c, glycosylated haemoglobin; FPG, fasting plasma glucose; SBP, systolic blood pressure; DBP, diastolic blood pressure.

### Unadjusted correlation between baseline data and T2DM

The results of univariate Cox proportional hazards models between baseline variables and T2DM were presented in **Table 2**. We found that male, age, BMI, WC, ALT, AST, GGT, TC, TG, HbA1c, FPG, SBP, DBP, fatty liver, smoking status (past or current) and alcohol consumption (heavy) (*P* < 0.05) were positively correlated with the risk of T2DM, and that HDL (*P* < 0.0001) was negatively correlated with the risk of T2DM.

**Table 2:** The unadjusted association between baseline variables and T2DM at the end of follow-up.

	T2DM	
	Hazard ratio (95% CI)	*P*-value
Men	2.52 (1.98, 3.21)	<0.0001
Age, yrs	1.06 (1.04, 1.07)	<0.0001
BMI, kg/m2	1.24 (1.22, 1.27)	<0.0001
WC, cm	1.09 (1.08, 1.10)	<0.0001
ALT, IU/L	1.01 (1.01, 1.01)	<0.0001
AST, IU/L	1.01 (1.01, 1.01)	<0.0001
GGT, IU/L	1.01 (1.01, 1.01)	<0.0001
TC, mg/dl	1.01 (1.01, 1.01)	<0.0001
HDL, mg/dl	0.95 (0.94, 0.96)	<0.0001
TG, mg/dl	1.01 (1.01, 1.01)	<0.0001
HbA1c, %	54.27 (39.49, 74.59)	<0.0001
FPG, mg/dl	1.20 (1.18, 1.22)	<0.0001
SBP, mmHg	1.03 (1.03, 1.04)	<0.0001
DBP, mmHg	1.05 (1.04, 1.06)	<0.0001
Fatty liver	7.02 (5.70, 8.63)	<0.0001
Smoking status		
Past	1.65 (1.26, 2.18)	0.0004
Current	2.58 (2.06, 3.24)	<0.0001
Alcohol consumption		
Light	0.90 (0.65, 1.26)	0.5508
Moderate	1.15 (0.82, 1.62)	0.424
Heavy	2.24 (1.54, 3.27)	<0.0001
Habit of exercise	0.76 (0.56, 1.02)	0.0641

Data are Hazard ratios and 95% CI.

T2DM, type 2 diabetes mellitus; BMI, body mass index; WC, waist circumference; ALT, alanine aminotransferase; AST, aspartate aminotransferase; GGT, gamma-glutamyl transferase; TC, total cholesterol; HDL, high-density lipoprotein cholesterol; TG, triglycerides; HbA1c, glycosylated haemoglobin; FPG, fasting plasma glucose; SBP, systolic blood pressure; DBP, diastolic blood pressure.

### Independent relation between ByG and T2DM

The results of the multiple Cox proportional hazards models are shown in **Table 3**. With the lowest tertile as a reference, unadjusted, minimally adjusted (adjusted for age and sex) and fully adjusted (adjusted for age, sex, ALT, GGT, TC, fatty liver, smoking status, alcohol consumption and habit of exercise) Cox regression analysis showed that the hazard ratios (HRs) for T2DM significantly increased as the tertiles of ByG increased. Moreover, multiple Cox regression analysis demonstrated that ByG per-SD change was positively correlated with the risk of T2DM (HR:2.57 in all populations; HR:1.97 in the normoglycemia cohort; HR:1.50 in the prediabetes cohort; all *P* < 0.0001).

**Table 3:** Associations of baseline ByG with incident T2DM.

	Crude model		Minimally model		Fully model	
	Hazard ratio (95%CI)	*P*-value	Hazard ratio (95%CI)	*P*-value	Hazard ratio (95%CI)	*P*-value
All populations						
ByG Per-SD increase	3.23 (2.92, 3.57)	<0.0001	3.35 (3.01, 3.74)	<0.0001	2.57 (2.26, 2.92)	<0.0001
ByG Tertiles						
T1	Reference		Reference		Reference	
T2	3.73 (2.15, 6.47)	<0.0001	3.34 (1.91, 5.84)	<0.0001	2.98 (1.69, 5.26)	0.0002
T3	19.27 (11.65, 31.87)	<0.0001	16.27(9.68,27.37)	<0.0001	9.25 (5.37, 15.96)	<0.0001
*P* for trend	<0.0001		<0.0001		<0.0001	
Normoglycemia						
ByG Per-SD increase	2.47 (2.02, 3.03)	<0.0001	2.52 (2.02, 3.15)	<0.0001	1.97 (1.53, 2.54)	<0.0001
ByG Tertiles						
T1	Reference		Reference		Reference	
T2	2.65 (1.23, 5.70)	0.0127	2.32 (1.06, 5.04)	0.0342	2.29 (1.04, 5.02)	0.0391
T3	6.05 (2.99, 12.24)	<0.0001	4.86 (2.32, 10.19)	<0.0001	2.95 (1.34, 6.51)	0.0073
*P* for trend	<0.0001		<0.0001		0.0086	
Prediabetes						
ByG Per-SD increase	1.83 (1.62, 2.07)	<0.0001	1.93 (1.70, 2.19)	<0.0001	1.50 (1.30, 1.74)	<0.0001
ByG Tertiles						
T1	Reference		Reference		Reference	
T2	2.03 (1.41, 2.93)	0.0001	2.03 (1.40, 2.95)	0.0002	1.48 (1.01, 2.18)	0.0452
T3	3.49 (2.48, 4.92)	<0.0001	3.68 (2.59, 5.22)	<0.0001	2.05 (1.40, 3.01)	0.0003
*P* for trend	<0.0001		<0.0001		0.0001	

Statistical analysis method used: cox regression analysis.

ByG, body mass index glucose; T2DM, type 2 diabetes mellitus.

Crude model adjusted for: none.

Minimally model adjusted for: sex; age.

Fully model adjusted for: sex; age; alanine aminotransferase; gamma-glutamyl transferase; total cholesterol; fatty liver; alcohol consumption; smoking status; habit of exercise.

**Figure 2:**
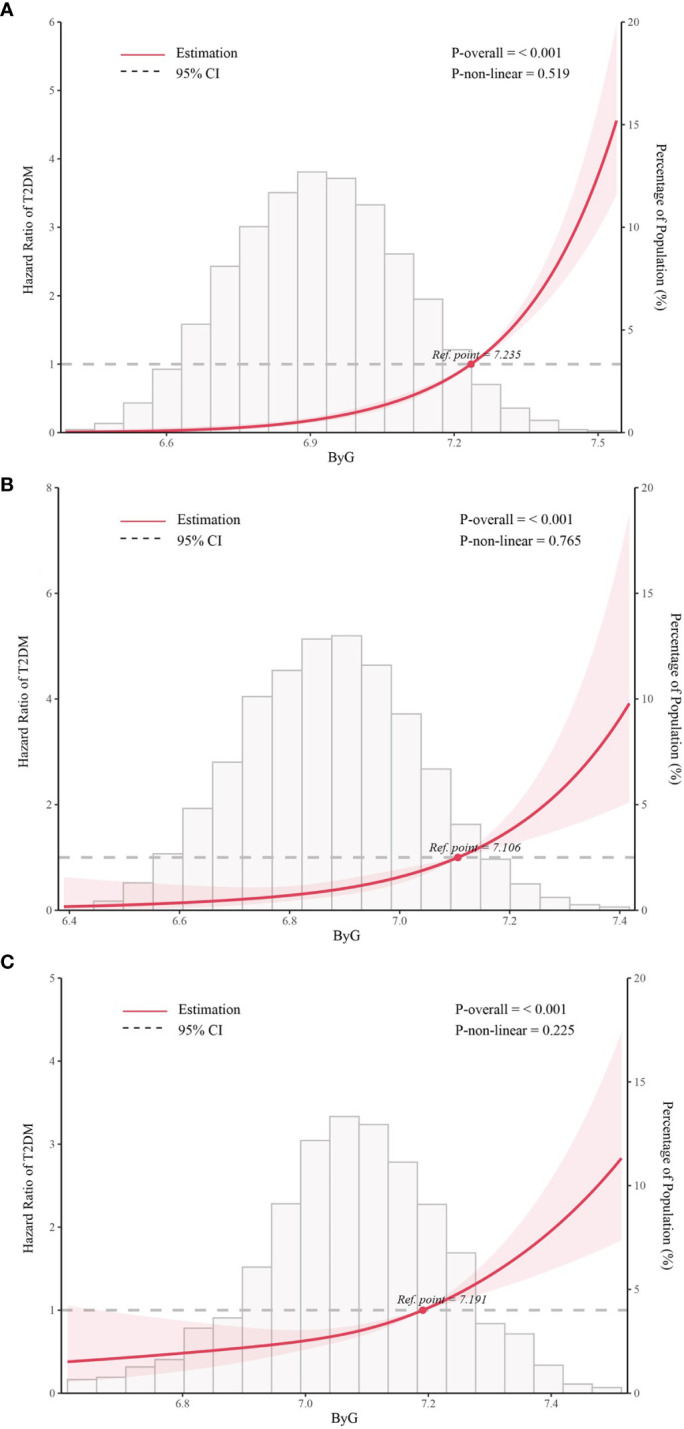
Association between ByG and T2DM in different populations. Data were fit by five knots restricted cubic spline (RCS). **(A)**. all populations, **(B)**. normoglycemia, **(C)**. prediabetes. ByG, body mass index glucose; T2DM, type 2 diabetes mellitus. Data were adjusted for sex, age, alanine aminotransferase, gamma-glutamyl transferase, total cholesterol, fatty liver, alcohol consumption, smoking status, habit of exercise.

We further assessed the potential nonlinear relationship between ByG and new-onset T2DM. The RCS analysis (**Figure 2**) showed a linear relationship between ByG and T2DM in all populations, normoglycemia and prediabetes (all *P* for nonlinearity > 0.05).

### Predictive efficacy of ByG for new-onset T2DM

**Figure 3:**
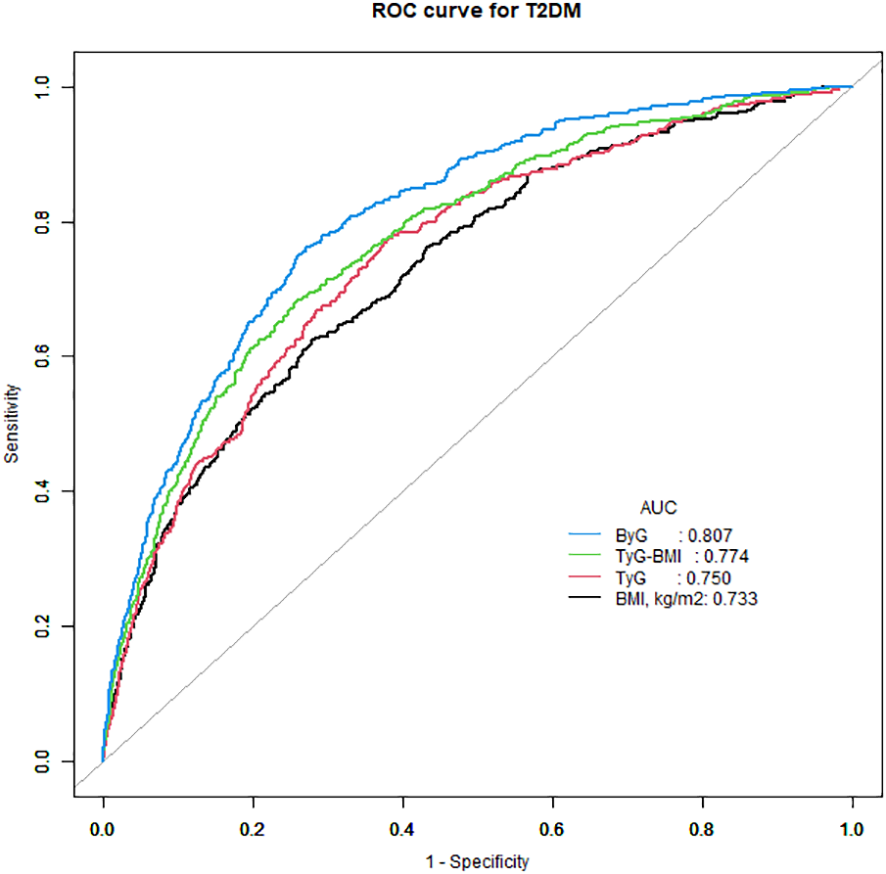
ROC curve analysis of the relationship between different indices and T2DM. T2DM, type 2 diabetes mellitus; TyG, triglyceride glucose; TyG-BMI, triglyceride glucose-body mass index; ByG, body mass index glucose.

ROC curve analysis was done to assess the effectiveness of ByG in predicting T2DM risk in all populations (**Figure 3**). The result showed that ByG (AUC 0.807) had the strongest predictive performance for T2DM compared with BMI (AUC 0.733), TyG (AUC 0.750) and TyG-WC (AUC 0.774). Consistent with this result, ByG was almost 1.23 times more informative about the risk of T2DM than TyG and TyG-BMI (**Table 4**).

**Table 4:** Relative informativeness of different indices for T2DM.

	Confounder adjusted χ² statistic	Informativeness*
TyG	512.4	100%
TyG-BMI	512.4	100%
ByG	632.2	123%

* Informativeness of the given measure (as indicated by the confounder-adjusted χ2 statistic relating it to T2DM), as a percentage of the informativeness of TyG.

T2DM, type 2 diabetes mellitus; TyG, triglyceride glucose; TyG-BMI, triglyceride glucose-body mass index; ByG, body mass index glucose.

Models adjusted for sex; age; alanine aminotransferase; gamma-glutamyl transferase; total cholesterol; fatty liver; alcohol consumption; smoking status; habit of exercise.

### Association between ByG and T2DM in subgroups

For each SD increase of ByG, the HRs for T2DM were 2.59 in females and 2.54 in males; 2.53 in non-fatty liver and 2.51 in fatty liver; 2.78 in never smokers, 1.99 in previous smokers and 2.69 in current smokers; 2.61 in never drinkers, 2.29 in light drinkers and 2.45 in moderate drinkers;2.48 in persons without habit of exercise and 3.68 in persons with habit of exercise in **Table 5**. No interaction between ByG and T2DM was observed between subgroups by grouping sex, fatty liver, smoking status (never, past, current), alcohol consumption (non, light, moderate, heavy), and habit of exercise in **Table 5** (all *P*_interaction_ > 0.05 or close to 0.05).

**Table 5:** Association of ByG with incident T2DM in subgroups.

	T2DM		
	No. of events	Hazard ratio (95% CI)	*P* for interaction
Sex			0.5021
Female	7034	2.59 (2.05, 3.28)	
Male	8430	2.54 (2.16, 2.97)	
Fatty liver			0.9235
No	12723	2.53 (2.07, 3.11)	
Yes	2741	2.51 (2.11, 2.98)	
Smoking status			0.2125
Never	9031	2.78 (2.28, 3.38)	
Past	2952	1.99 (1.42, 2.78)	
Current	3481	2.69 (2.18, 3.30)	
Alcohol consumption			0.9129
No	11805	2.61 (2.25, 3.02)	
Light	1758	2.29 (1.51, 3.46)	
Moderate	1360	2.45 (1.51, 3.99)	
Heavy	541	-^a^	
Habit of exercise			0.0473
No	12755	2.48 (2.17, 2.84)	
Yes	2709	3.68 (2.49, 5.46)	

Data were adjusted for sex; age; alanine aminotransferase; gamma-glutamyl transferase; total cholesterol; fatty liver; alcohol consumption; smoking status; habit of exercise.

ByG, body mass index glucose; T2DM, type 2 diabetes mellitus.

a The model failed because of the small sample size.

## Discussion

### Principal findings

Here, we provided for the first time evidence that ByG was independently positively associated with T2DM in Japan. Moreover, these associations were independent of age, sex, ALT, GGT, TC, fatty liver, smoking status, alcohol consumption and habit of exercises. The present study may suggest that ByG was a better predictor of T2DM than BMI, TyG and TyG-BMI.

### Comparison with other studies

Several screening indicators that predict high risk of future T2DM have been previously reported. Given the high cost of time and money, oral glucose tolerance test is seldom used for routine testing. A Swedish case-referent study showed that the combination of HbA1c, FPG and BMI was effective in screening for individuals at risk of future clinical diagnosis of T2DM (28). Both Japanese and Chinese studies suggested that the combined measurement of FPG and HbA1c was effective for predicting T2DM (29, 30). A study in the Finnish population revealed that these individuals can be identified early by knowledge of FPG, BMI, and family history of diabetes (31). The American Diabetes Association diabetes risk test, as a simple and inexpensive tool to identify individuals at high risk for T2DM, had highlighted the importance of BMI in predicting the risk of T2DM (32). Different from previous studies, our study was the first to demonstrate a positive association between the combination of BMI and FPG and T2DM and suggested that ByG may be used to identify people who were at high risk for developing T2DM.

IR was one of the important factors affecting various metabolic diseases. Thus, identifying IR was critically important for early diagnosis, prevention, and personalized therapy. The TyG was a new and economical index for IR evaluation. In recent years, the role of TyG in the evaluation of diabetes, non-alcoholic fatty liver disease and other common clinical diseases has been confirmed to a certain extent (33–35), which has become a new research direction. A meta-analysis in 2020 demonstrated that TyG was a predictor of T2DM development (17). Another meta-analysis in 2021 assessed the dose-response relationship between TyG and the incidence of T2DM (36). It has been found that a high TyG was associated with the incidence of T2DM and the relationship between them was non-linear (36). Other studies on IR and diabetes indicated that the combined application of BMI with TyG (TyG-BMI) showed a significant increase in the area under the curve and its discrimination accuracy was higher than TyG (19, 20). This meant that BMI was essentially important to predict diabetes risk. Given that TG levels were affected by race and steroid hormones (37), we created a new index - ByG, which linked together BMI and FPG. Importantly, ByG was much easier to compute and utilize than TyG-BMI. Compared with TyG and TyG-BMI, ByG showed a better performance in identifying T2DM. More future studies are needed to gain more insights about ByG.

### Meaning of the study

Many studies have pointed to a strong relationship between obesity and diabetes (5, 38, 39). Also, obesity was closely related to high blood sugar (40). Moreover, reactive oxygen species, mainly derived from adipose tissue, may cause multiple metabolic disorders, including obesity-related IR and T2DM (41). BMI, calculated from weight and height, represented general obesity. In addition to obesity, elevated FPG levels have also been shown to be an independent risk factor for the development of T2DM (42). Our results showed that ByG, the combination of obesity and FPG, was a predictor of diabetes. The dominance of ByG may be due to the well-verified roles of FPG and obesity in the development of IR and diabetes. Elevated glucose concentrations have a toxic effect upon beta cells by increasing reactive oxygen species (43). Altogether, these results suggested that glucotoxicity and lipotoxicity played pivotal roles for pathogenesis of diabetes.

Furthermore, BMI was a cheap, easy-to-operate and non-invasive compared to TG. Advantageously, an accurate scale enabled real-time measurement of BMI at home. ByG simplified the calculation of TyG-BMI. Due to its convenience and simplification, ByG was not only suitable for large-scale health assessment, but also for individuals’ evaluation of themselves.

### Strength and limitations of this study

This analysis had important strengths. The large size and long follow-up duration of the two cohort studies strengthened the credibility of the results. More importantly, more confounding factors were adjusted to increase the reliability of the results compared with previous studies. Besides, because the data come from Japan, the results were more instructive to the Japanese. It was interesting to notice that ByG would be a new predictor of diabetes. In addition, ByG can serve as a reference biomarker for T2DM during follow-up.

Several limitations may exist in this study. Firstly, as a single-center cohort study in Japan, the results may not be directly applicable to other regions and ethnicities. The generality of the findings needed to be confirmed across regions and ethnic groups. Then, the prevalence of diabetes may be inaccurate due to the lack of oral glucose tolerance tests. Lastly, because the research data came from an existing database, unmeasured confounding factors may not fully be resolved. We were unable to obtain potentially important information that may influence T2DM, such as family history of diabetes, history of gestational diabetes, therapy and main comorbidities (in particular obesity).

## Conclusions

The study in Japanese population have first found evidence that higher tertiles of ByG were associated with a higher risk of T2DM. ByG assessment may have important implications for identifying individuals at increased risk of T2DM in the clinic. Future prospective clinical studies should focus on the underlying mechanisms of these associations that have not been fully elucidated.

## Data availability statement

Publicly available datasets were analyzed in this study. This data can be found here: https://datadryad.org/stash/dataset/doi:10.5061%2Fdryad.8q0p192.

## Ethics statement

The studies involving humans were approved by the Ethics Committee of Murakami Memorial Hospital in Japan. The studies were conducted in accordance with the local legislation and institutional requirements. The participants provided their written informed consent to participate in this study.

## Author contributions

XZ: Data curation, Resources, Writing – original draft, Writing – review & editing. TY: Data curation, Writing – original draft, Writing – review & editing. BS: Methodology, Writing – original draft. HF: Methodology, Writing – original draft. TL: Writing – review & editing. GG: Writing – review & editing. KW: Writing – review & editing. WL: Conceptualization, Project administration, Writing – review & editing. CL: Conceptualization, Funding acquisition, Project administration, Writing – review & editing.
